# Vitamin D supplementation to prevent acute respiratory tract infections: systematic review and meta-analysis of individual participant data

**DOI:** 10.1136/bmj.i6583

**Published:** 2017-02-15

**Authors:** Adrian R Martineau, David A Jolliffe, Richard L Hooper, Lauren Greenberg, John F Aloia, Peter Bergman, Gal Dubnov-Raz, Susanna Esposito, Davaasambuu Ganmaa, Adit A Ginde, Emma C Goodall, Cameron C Grant, Christopher J Griffiths, Wim Janssens, Ilkka Laaksi, Semira Manaseki-Holland, David Mauger, David R Murdoch, Rachel Neale, Judy R Rees, Steve Simpson, Iwona Stelmach, Geeta Trilok Kumar, Mitsuyoshi Urashima, Carlos A Camargo

**Affiliations:** 1 Centre for Primary Care and Public Health, Blizard Institute, Barts and The London School of Medicine and Dentistry, Queen Mary University of London, London E1 2AB, UK; 2 Asthma UK Centre for Applied Research, Blizard Institute, Queen Mary University of London, London, UK; 3 Bone Mineral Research Center, Winthrop University Hospital, Mineola, NY, USA; 4 Department of Laboratory Medicine, Karolinska Institutet, Stockholm, Sweden; 5 Department of Exercise, Lifestyle and Nutrition Clinic, Edmond and Lily Safra Children’s Hospital, Tel Hashomer, Israel; 6 Pediatric Highly Intensive Care Unit, Fondazione IRCCS Ca’ Granda Ospedale Maggiore Policlinico, Università degli Studi di Milano, Milan, Italy; 7 Department of Nutrition, Harvard School of Public Health, Boston, MA, USA; 8 Department of Emergency Medicine, University of Colorado School of Medicine, Aurora, CO, USA; 9 Department of Clinical Epidemiology and Biostatistics, McMaster University, Hamilton, Ontario, Canada; 10 Department of Paediatrics: Child & Youth Health, Faculty of Medical and Health Sciences, University of Auckland, Auckland, New Zealand; 11 MRC and Asthma UK Centre in Allergic Mechanisms of Asthma, Blizard Institute, Queen Mary University of London, London, UK; 12 Universitair ziekenhuis Leuven, Leuven, Belgium; 13 Tampere School of Public Health, University of Tampere, Tampere, Finland; 14 Department of Public Health, Epidemiology and Biostatistics, Institute of Applied Health Sciences, College of Medical and Dental Sciences, University of Birmingham, Birmingham, UK; 15 Department of Statistics, The Pennsylvania State University, Hershey, PA, USA; 16 Department of Pathology, University of Otago, Christchurch, New Zealand; 17 QIMR Berghofer Medical Research Institute, Queensland, Australia; 18 Department of Epidemiology, Geisel School of Medicine at Dartmouth, Lebanon, NH, USA; 19 Menzies Institute for Medical Research, University of Tasmania, Hobart, Australia; 20 Department of Pediatrics and Allergy, Medical University of Lodz, Lodz, Poland; 21 Institute of Home Economics, University of Delhi, New Delhi, India; 22 Division of Molecular Epidemiology, Jikei University School of Medicine, Tokyo, Japan; 23 Department of Emergency Medicine, Massachusetts General Hospital, Harvard Medical School, Boston, MA, USA

## Abstract

**Objectives** To assess the overall effect of vitamin D supplementation on risk of acute respiratory tract infection, and to identify factors modifying this effect.

**Design** Systematic review and meta-analysis of individual participant data (IPD) from randomised controlled trials.

**Data sources** Medline, Embase, the Cochrane Central Register of Controlled Trials, Web of Science, ClinicalTrials.gov, and the International Standard Randomised Controlled Trials Number registry from inception to December 2015.

**Eligibility criteria for study selection** Randomised, double blind, placebo controlled trials of supplementation with vitamin D_3_ or vitamin D_2_ of any duration were eligible for inclusion if they had been approved by a research ethics committee and if data on incidence of acute respiratory tract infection were collected prospectively and prespecified as an efficacy outcome.

**Results** 25 eligible randomised controlled trials (total 11 321 participants, aged 0 to 95 years) were identified. IPD were obtained for 10 933 (96.6%) participants. Vitamin D supplementation reduced the risk of acute respiratory tract infection among all participants (adjusted odds ratio 0.88, 95% confidence interval 0.81 to 0.96; P for heterogeneity <0.001). In subgroup analysis, protective effects were seen in those receiving daily or weekly vitamin D without additional bolus doses (adjusted odds ratio 0.81, 0.72 to 0.91) but not in those receiving one or more bolus doses (adjusted odds ratio 0.97, 0.86 to 1.10; P for interaction=0.05). Among those receiving daily or weekly vitamin D, protective effects were stronger in those with baseline 25-hydroxyvitamin D levels <25 nmol/L (adjusted odds ratio 0.30, 0.17 to 0.53) than in those with baseline 25-hydroxyvitamin D levels ≥25 nmol/L (adjusted odds ratio 0.75, 0.60 to 0.95; P for interaction=0.006). Vitamin D did not influence the proportion of participants experiencing at least one serious adverse event (adjusted odds ratio 0.98, 0.80 to 1.20, P=0.83). The body of evidence contributing to these analyses was assessed as being of high quality.

**Conclusions** Vitamin D supplementation was safe and it protected against acute respiratory tract infection overall. Patients who were very vitamin D deficient and those not receiving bolus doses experienced the most benefit.

**Systematic review registration** PROSPERO CRD42014013953.

## Introduction

Acute respiratory tract infections are a major cause of global morbidity and mortality and are responsible for 10% of ambulatory and emergency department visits in the USA^1^ and an estimated 2.65 million deaths worldwide in 2013.^2^ Observational studies report consistent independent associations between low serum concentrations of 25-hydroxyvitamin D (the major circulating vitamin D metabolite) and susceptibility to acute respiratory tract infection.^3^ ^4^ 25-hydroxyvitamin D supports induction of antimicrobial peptides in response to both viral and bacterial stimuli,^5^ ^6^ ^7^ suggesting a potential mechanism by which vitamin D inducible protection against respiratory pathogens might be mediated. Vitamin D metabolites have also been reported to induce other innate antimicrobial effector mechanisms, including induction of autophagy and synthesis of reactive nitrogen intermediates and reactive oxygen intermediates.^8^ These epidemiological and in vitro data have prompted numerous randomised controlled trials to determine whether vitamin D supplementation can decrease the risk of acute respiratory tract infection. A total of five aggregate data meta-analyses incorporating data from up to 15 primary trials have been conducted to date, of which two report statistically significant protective effects^9^ ^10^ and three report no statistically significant effects.^11^ ^12^ ^13^ All but one of these aggregate data meta-analyses^11^ reported statistically significant heterogeneity of effect between primary trials.

This heterogeneity might have arisen as a result of variation in participant characteristics and dosing regimens between trials, either of which may modify the effects of vitamin D supplementation on immunity to respiratory pathogens.^14^ People with chronic obstructive pulmonary disease who have lower baseline vitamin D status have been reported to derive greater clinical benefit from supplementation than those with higher baseline status,^15^ ^16^ and participant characteristics such as age and body mass index have been reported to modify the 25-hydroxyvitamin D response to vitamin D supplementation.^17^ ^18^ Treatment with large boluses of vitamin D has been associated with reduced efficacy for non-classic effects,^9^ and in some cases an increased risk of adverse outcomes.^19^ While study level factors are amenable to exploration through aggregate data meta-analysis of published data, potential effect modifiers operating at an individual level, such as baseline vitamin D status, can only be explored using individual participant data (IPD) meta-analysis. This is because subgroups are not consistently disaggregated in trial reports, and adjustments for potential confounders cannot be applied similarly across trials.^20^ To identify factors that might explain the observed heterogeneity of results from randomised controlled trials, we undertook an IPD meta-analysis based on all 25 randomised controlled trials of vitamin D supplementation for prevention of acute respiratory tract infection that were completed up to the end of December 2015.

## Methods

### Protocol and registration

The methods were prespecified in a protocol that was registered with the PROSPERO International Prospective Register of Systematic Reviews (www.crd.york.ac.uk/PROSPERO/display_record.asp?ID=CRD42014013953). Approval by a research ethics committee to conduct this meta-analysis was not required in the UK; local ethical permission to contribute deidentified IPD from primary trials was required and obtained for studies by Camargo et al^21^ (the ethics review committee of the Mongolian Ministry of Health), Murdoch et al^22^ (Southern Health and Disability Ethics Committee, reference URB/09/10/050/AM02), Rees et al^23^ (Committee for the Protection of Human Subjects, Dartmouth College, USA; protocol No 24381), Tachimoto et al^24^ (ethics committee of the Jikei University School of Medicine, reference 26-333: 7839), Tran et al^25^ (QIMR Berghofer Medical Research Institute human research ethics committee, P1570), and Urashima et al^26^ ^27^ (ethics committee of the Jikei University School of Medicine, reference 26-333: 7839).

### Patient and public involvement

Two patient and public involvement representatives were involved in development of the research questions and the choice of outcome measures specified in the study protocol. They were not involved in patient recruitment, since this is a meta-analysis of completed studies. Data relating to the burden of the intervention on participants’ quality of life and health were not meta-analysed. Where possible, results of this systematic review and meta-analysis will be disseminated to individual participants through the principal investigators of each trial.

### Eligibility criteria

Randomised, double blind, placebo controlled trials of supplementation with vitamin D_3_ or vitamin D_2_ of any duration were eligible for inclusion if they had been approved by a research ethics committee and if data on incidence of acute respiratory tract infection were collected prospectively and prespecified as an efficacy outcome. The last requirement was imposed to minimise misclassification bias (prospectively designed instruments to capture acute respiratory tract infection events were deemed more likely to be sensitive and specific for this outcome). We excluded studies reporting results of long term follow-up of primary randomised controlled trials.

### Study identification and selection

Two investigators (ARM and DAJ) searched Medline, Embase, the Cochrane Central Register of Controlled Trials (CENTRAL), Web of Science, ClinicalTrials.gov, and the International Standard Randomized Controlled Trials Number (ISRCTN) registry using the electronic search strategies described in the supplementary material. Searches were regularly updated up to, and including, 31 December 2015. No language restrictions were imposed. These searches were supplemented by searches of review articles and reference lists of trial publications. Collaborators were asked if they knew of any additional trials. Two investigators (ARM and CAC) determined which trials met the eligibility criteria.

### Data collection processes

IPD were requested from the principal investigator for each eligible trial, and the terms of collaboration were specified in a data transfer agreement, signed by representatives of the data provider and the recipient (Queen Mary University of London). Data were deidentified at source before transfer by email. On receipt, three investigators (DAJ, RLH, and LG) assessed data integrity by performing internal consistency checks and by attempting to replicate results of the analysis for incidence of acute respiratory tract infection where this was published in the trial report. Study authors were contacted to provide missing data and to resolve queries arising from these integrity checks. Once queries had been resolved, clean data were uploaded to the main study database, which was held in STATA IC v12 (College Station, TX).

Data relating to study characteristics were extracted for the following variables: setting, eligibility criteria, details of intervention and control regimens, study duration, and case definitions for acute respiratory tract infection. IPD were extracted for the following variables, where available: baseline data were requested for age, sex, cluster identifier (cluster randomised trials only), racial or ethnic origin, influenza vaccination status, history of asthma, history of chronic obstructive pulmonary disease, body weight, height (adults and children able to stand) or length (infants), serum 25-hydroxyvitamin D concentration, study allocation (vitamin D versus placebo), and details of any stratification or minimisation variables. Follow-up data were requested for total number of acute respiratory tract infections (upper or lower), upper respiratory tract infections, and lower respiratory tract infections experienced during the trial; time from first dose of study drug to first acute respiratory tract infection (upper or lower), upper respiratory tract infection, or lower respiratory tract infection if applicable; total number of courses of antibiotics taken for acute respiratory tract infection during the trial; total number of days off work or school due to symptoms of acute respiratory tract infection during the trial; serum 25-hydroxyvitamin D concentration at final follow-up; duration of follow-up; number and nature of serious adverse events; number of potential adverse reactions (incident hypercalcaemia or renal stones); and participant status at end of the trial (completed, withdrew, lost to follow-up, died).

### Risk of bias assessment for individual studies

We used the Cochrane Collaboration risk of bias tool^28^ to assess sequence generation; allocation concealment; blinding of participants, staff, and outcome assessors; completeness of outcome data; and evidence of selective outcome reporting and other potential threats to validity. Two investigators (ARM and DAJ) independently assessed study quality, except for the three trials by Martineau and colleagues, which were assessed by CAC. Discrepancies were resolved by consensus.

### Definition of outcomes

The primary outcome of the meta-analysis was incidence of acute respiratory tract infection, incorporating events classified as upper respiratory tract infection, lower respiratory tract infection, and acute respiratory tract infection of unclassified location (ie, infection of the upper respiratory tract or lower respiratory tract, or both). Secondary outcomes were incidence of upper and lower respiratory tract infections, analysed separately; incidence of emergency department attendance or hospital admission, or both for acute respiratory tract infection; use of antimicrobials for treatment of acute respiratory tract infection; absence from work or school due to acute respiratory tract infection; incidence and nature of serious adverse events; incidence of potential adverse reactions to vitamin D (hypercalcaemia or renal stones); and mortality (acute respiratory tract infection related and all cause).

### Synthesis methods

LG and RLH analysed the data. Our IPD meta-analysis approach followed published guidelines.^20^ Initially we reanalysed all studies separately; the original authors were asked to confirm the accuracy of this reanalysis where it had been performed previously, and any discrepancies were resolved. Then we performed both one step and two step IPD meta-analysis for each outcome separately using a random effects model adjusted for age, sex, and study duration to obtain the pooled intervention effect with a 95% confidence interval. We did not adjust for other covariates because missing values for some participants would have led to their exclusion from statistical analyses. In the one step approach, we modelled IPD from all studies simultaneously while accounting for the clustering of participants within studies. In the two step approach we first analysed IPD for each separate study independently to produce an estimate of the treatment effect for that study; we then synthesised these data in a second step.^20^ For the one step IPD meta-analysis we assessed heterogeneity by calculation of the standard deviation of random effects; for the two step IPD meta-analysis we summarised heterogeneity using the I^2^ statistic. We calculated the number needed to treat to prevent one person from having any acute respiratory tract infection (NNT) using the Visual Rx NNT calculator (www.nntonline.net/visualrx/), where meta-analysis of dichotomous outcomes revealed a statistically significant beneficial effect of allocation to vitamin D compared with placebo.

### Exploration of variation in effects

To explore the causes of heterogeneity and identify factors modifying the effects of vitamin D supplementation, we performed prespecified subgroup analyses by extending the one step meta-analysis framework to include treatment-covariate interaction terms. Subgroups were defined according to baseline vitamin D status (serum 25-hydroxyvitamin D <25 *v* ≥25 nmol/L), vitamin D dosing regimen (daily or weekly without bolus dosing versus a regimen including at least one bolus dose of at least 30 000 IU vitamin D), dose size (daily equivalent <800 IU, 800-1999 IU, ≥2000 IU), age (≤1 year, 1.1-15.9 years, 16-65 years, >65 years), body mass index (<25 *v* ≥25), and presence compared with absence of asthma, chronic obstructive pulmonary disease, and previous influenza vaccination. To ensure that reported subgroup effects were independent, we adjusted interaction analyses for potential confounders (age, sex, and study duration). The 25 nmol/L cut-off for baseline 25-hydroxyvitamin D concentration in subgroup analyses was selected on the grounds that it is the threshold for vitamin D deficiency defined by the UK Department of Health,^29^ and the level below which participants in clinical trials have experienced the most consistent benefits of supplementation.^30^ We also performed an exploratory analysis investigating effects in subgroups defined using the 50 nmol/L and 75 nmol/L cut-offs for baseline circulating 25-hydroxyvitamin D concentration, because observational studies have reported that less profound states of vitamin D deficiency may also associate independently with an increased risk of acute respiratory tract infection.^31^ ^32^ To minimise the chance of type 1 error arising from multiple analyses, we inferred statistical significance for subgroup analyses only where P values for treatment-covariate interaction terms were <0.05.

### Quality assessment across studies

For the primary analysis we investigated the likelihood of publication bias through the construction of a contour enhanced funnel plot.^33^ We used the five GRADE considerations (study limitations, consistency of effect, imprecision, indirectness, and publication bias)^34^ to assess the quality of the body of evidence contributing to analyses of the primary efficacy outcome and major safety outcome of our meta-analysis (see supplementary table S3).

### Additional analyses

We conducted sensitivity analyses excluding IPD from trials where acute respiratory tract infection was a secondary outcome (as opposed to a primary or co-primary outcome), and where risk of bias was assessed as being unclear. We also conducted a responder analysis in participants randomised to the intervention arm of included studies for whom end study data on 25-hydroxyvitamin D were available, comparing risk of acute respiratory tract infection in those who attained a serum level of 75 nmol/L or more compared with those who did not.

## Results

### Study selection and IPD obtained

Our search identified 532 unique studies that were assessed for eligibility; of these, 25 studies with a total of 11 321 randomised participants fulfilled the eligibility criteria (fig 1). IPD were sought and obtained for all 25 studies. Outcome data for the primary analysis of proportion of participants experiencing at least one acute respiratory tract infection were obtained for 10 933 (96.6%) of the randomised participants.

**Figure f1:**
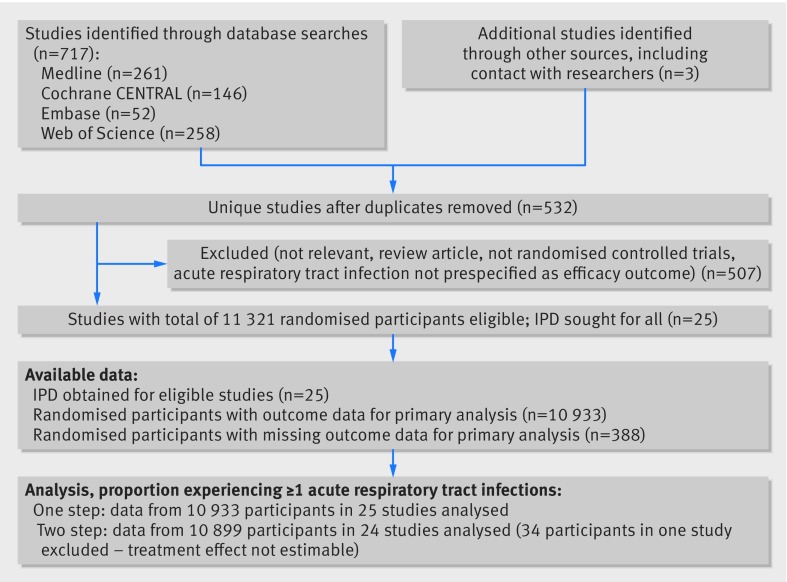
**Fig 1** Flow of study selection. IPD=individual participant data

### Study and participant characteristics

Table 1 presents the characteristics of eligible studies and their participants. Trials were conducted in 14 countries on four continents and enrolled participants of both sexes from birth to 95 years of age. Baseline serum 25-hydroxyvitamin D concentrations were determined in 19/25 trials: mean baseline concentration ranged from 18.9 to 88.9 nmol/L. Baseline characteristics of participants randomised to intervention and control were similar (see supplementary table S1). All studies administered oral vitamin D_3_ to participants in the intervention arm: this was given as bolus doses every month to every three months in seven studies, weekly doses in three studies, a daily dose in 12 studies, and a combination of bolus and daily doses in three studies. Study duration ranged from seven weeks to 1.5 years. Incidence of acute respiratory tract infection was the primary or co-primary outcome for 14 studies and a secondary outcome for 11 studies.

**Table 1 tbl1:** Characteristics of the 25 eligible trials and their participants

Reference	Setting (study duration)	Participants (male:female)	Mean (SD) age, years (range)	25(OH)D			No in intervention:control group	Oral dose of vitamin D_3_	ARTI		No entering primary analysis/No randomised (%)
				Assay, EQA scheme	Mean (SD) baseline level, nmol/L (range)	Baseline level <25 nmol/L (%)			Definition	Outcome type	
Li-Ng 2009^41^	USA (3 months)	Healthy adults (34:128)	57.9 (13.6) (21.4-80.6)	RIA (DiaSorin), DEQAS	63.7 (25.5) (16.0-156.0)	3/150 (2.0)	84:78	50 µg daily, placebo	URTI: ≥2 URTI symptoms in absence of allergy symptoms	Primary	157/162 (96.9)
Urashima 2010^27^	Japan (4 months)	Schoolchildren (242:188)	10.2 (2.3) (6.0-15.0)	--	ND	--	217:213	30 µg daily, placebo	URTI: influenza A/B diagnosed by RIDT or RIDT-negative ILI	Primary	334/430 (77.7)
Manaseki-Holland 2010^42^	Afghanistan (3 months)	Preschool children with pneumonia (257:196)	1.1 (0.8) (0.1-3.3)	--	ND	--	224:229	2.5 mg bolus once, placebo	LRTI: repeat episode of pneumonia—age-specific tachypnoea without wheeze	Secondary	453/453 (100.0)
Laaksi 2010^37^	Finland (6 months)	Military conscripts (164:0)	19.1 (0.6) (18.0-21.0)	EIA (IDS OCTEIA)	75.9 (18.7) (41.9-129.0)	0/73 (0.0)	80:84	10 µg daily, placebo	ARTI: medical record diagnosis	Primary	164/164 (100.0)
Majak 2011^43^	Poland (6 months)	Children with asthma (32:16)	10.9 (3.3) (6.0-17.0)	RIA (BioSource Europe), RIQAS	88.9 (38.2) (31.5-184.7)	0/48 (0.0)	24:24	12.5 µg daily, placebo	ARTI: self report	Secondary	48/48 (100.0)
Trilok-Kumar 2011^44^	India (6 months)	Low birthweight infants (970:1109)	0.1 (0.0) (0.0-0.3)	--	ND	ND	1039:1040	35 µg weekly, placebo	ARTI: medical record diagnosis of events resulting in hospital admission	Secondary	2064/2079 (99.3)
Lehouck 2012^15^	Belgium (1 year)	Adults with COPD (145:37)	67.9 (8.3) (48.0-86.0)	RIA (Diasorin), DEQAS	49.8 (29.2) (9.0-159.7)	31/182 (17.0)	91:91	2.5 mg bolus monthly, placebo	URTI: self report	Secondary	175/182 (96.2)
Manaseki-Holland 2012^35^	Afghanistan (1.5 years)	Infants (1591:1455)	0.5 (0.3) (0.0-1.0)	--	ND	ND	1524:1522	2.5 mg bolus 3-monthly, placebo	LRTI: pneumonia confirmed by chest radiography	Primary	3011/3046 (98.9)
Camargo 2012^21^	Mongolia (7 weeks)	3rd/4th grade schoolchildren (129:118)	10.0 (0.9) (7.0-12.7)	LC-MS/MS, DEQAS	18.9 (9.7) (3.3-61.2)	192/245 (78.4)	143:104	7.5 µg daily, placebo	ARTI: parent reported “chest infections or colds”	Secondary	244/247 (98.8)
Murdoch 2012^22^	New Zealand (1.5 years)	Healthy adults (81:241)	48.1 (9.7) (18.0-67.6)	LC-MS/MS, DEQAS	72.1 (22.1) (13.0-142.0)	5/322 (1.6)	161:161	2×5 mg bolus monthly then 2.5 mg bolus monthly, placebo	URTI: assessed with symptom score	Primary	322/322 (100.0)
Bergman 2012^45^	Sweden (1 year)	Adults with increased susceptibility to ARTI (38:102)	53.1 (13.1) (20.0-77.0)	CLA (DiaSorin), DEQAS	49.3 (23.2) (8.0-135.0)	15/131 (11.45)	70:70	100 µg daily, placebo	URTI: assessed with symptom score	Secondary	124/140 (88.6)
Marchisio 2013^46^	Italy (6 months)	Children with recurrent acute otitis media (64:52)	2.8 (1.0) (1.3-4.8)	CLA (DiaSorin), ISO9001	65.3 (17.3) (24.7-120.6)	2/116 (1.7)	58:58	25 µg daily, placebo	URTI: doctor diagnosed acute otitis media	Primary	116/116 (100.0)
Rees 2013^23^	USA (13 months, average)	Adults with previous colorectal adenoma (438:321*)	61.2 (6.6) (47.1-77.9)	RIA (IDS), DEQAS	62.5 (21.3) (30.2-171.6)	0/759 (0.0)	399:360	25 µg daily, placebo	URTI: assessed from daily symptom diary	Secondary	759/759 (100.0)
Tran 2014^25^	Australia (1 year)	Healthy older adults (343:301)	71.7 (6.9) (60.3-85.2)	CLA (DiaSorin), DEQAS	41.7 (13.5) (12.6-105.0)	66/643 (10.3)	430:214	0.75 mg bolus *v* 1.5 mg bolus monthly, placebo	URTI: self reported cold	Secondary	594/644 (92.2)
Goodall 2014^47^	Canada (8 weeks)	Healthy university students (218:382)	19.6 (2.2) (17.0-33.0)	--	ND	--	300:300	0.25 mg weekly (factorial with gargling), placebo	URTI: self reported cold	Primary	492/600 (82.0)
Urashima 2014^26^	Japan (2 months)	High school students (162:85)	16.5 (1.0) (15.0-18.0)	--	ND	--	148:99	50 µg daily, placebo	URTI: influenza A diagnosed by RIDT or RIDT negative ILI	Primary	247/247 (100.0)
Grant 2014^48^	New Zealand (9 months: 3 months in pregnancy + 6 months in infancy)	Pregnant women and offspring (0:260 (mothers) 121:128 (offspring))	unborn	LC-MS/MS, DEQAS	54.8 (25.8) (8.0-128.0)	30/200 (15.0)	173:87 (mothers) 164:85 (offspring)	Mothers: 25 µg *v* 50 µg daily Infants: 10 µg *v* 20 µg daily, placebo	ARTI: doctor diagnosed ARTI precipitating primary care consultation	Secondary	236/260 (90.8)
Martineau 2015a^16^ (ViDiCO)	UK (1 year)	Adults with COPD (144:96)	64.7 (8.5) (40.0-85.0)	LC-MS/MS, DEQAS	46.1 (25.7) (0.0-160.0)	50/240 (20.8)	122:118	3 mg bolus 2-monthly, placebo	URTI: assessed from daily symptom diary	Coprimary	240/240 (100.0)
Martineau 2015b^49^ (ViDiAs)	UK (1 year)	Adults with asthma (109:141)	47.9 (14.4) (16.0-78.0)	LC-MS/MS, DEQAS	49.6 (24.7) (0.0-139.0)	36/250 (14.4)	125:125	3 mg bolus 2-monthly, placebo	URTI: assessed from daily symptom diary	Coprimary	250/250 (100.0)
Martineau 2015c^50^ (ViDiFlu)	UK (1 year)	Older adults and their carers (82:158)	67.1 (13.0) (21.4-94.0)	LC-MS/MS, DEQAS	42.9 (23.0) (0.0-128.0)	60/240 (25.0)	137:103	Older adults: 2.4 mg bolus 2-monthly+10 µg daily. Carers: 3 mg 2-monthly, older adults: placebo+10 µg daily. Carers: placebo	URTI and LRTI, both assessed from daily symptom diary	Coprimary	240/240 (100.0)
Simpson 2015^51^	Australia (17 weeks)	Healthy adults (14:20)	32.2 (12.2) (18.0-52.0)	LC-MS/MS, DEQAS	67.9 (23.0) (32.0-132.0)	0/33 (0.0)	18:16	0.5 mg weekly, placebo	ARTI assessed with symptom score	Primary	34/34 (100.0)
Dubnov-Raz 2015^36^	Israel (12 weeks)	Adolescent swimmers with vitamin D insufficiency (34:20)	15.2 (1.6) (12.9-18.6)	RIA (DiaSorin), DEQAS	60.4 (11.9) (28.0-74.6)	0/54 (0.0)	27:27	50 µg daily, placebo	URTI assessed with symptom score	Primary	25/54 (46.3)
Denlinger 2016^52^	USA (28 weeks)	Adults with asthma (130:278)	39.2 (12.9) (18.0-85.0)	CLA (DiaSorin), VDSP	47.0 (16.9) (10.0-74.6)	55/408 (13.5)	201:207	2.5 mg bolus then 100 µg daily, placebo	URTI assessed with symptom score	Secondary	408/408 (100.0)
Tachimoto 2016^24^	Japan (6 months)	Children with asthma (50:39)	9.9 (2.3) (6.0-15.0)	RIA (DiaSorin), CAP	74.9 (24.6) (20.0-187.2)	1/89 (1.1)	54:35	20 µg daily, first 2 months, placebo	URTI: assessed with symptom score	Secondary	89/89 (100.0)
Ginde, 2016^53^	USA (1 year)	Older care home residents (45:62)	80.7 (9.9) (60.0-95.0)	LC-MS/MS, VDSP	57.3 (22.7) (11.7-106.1)	12/107 (11.2)	55:52	2.5 mg bolus monthly+≤25 µg per day equivalent, placebo+10-25 µg per day equivalent	ARTI: medical record diagnosis	Primary	107/107 (100.0)

25(OH)D=25-hydroxyvitamin D; RIDT=rapid influenza diagnostic test; COPD=chronic obstructive pulmonary disease; D_3_, vitamin D_3_ (cholecalciferol); ARTI=acute respiratory tract infection; CAP=College of American Pathologists; CLA=chemiluminescent assay; DEQAS=Vitamin D External Quality Assessment Scheme; EIA=enzyme immunoassay; EQA=external quality assessment; LC-MS/MS=liquid chromatography tandem-mass spectrometry; RIA=radioimmunoassay; URTI=upper respiratory tract infection; LRTI=lower respiratory tract infection; ILI=influenza-like illness; RIQAS=Randox International Quality Assessment Scheme; VDSP=Vitamin D Standardisation Program of the Office of Dietary Supplements, National Institutes of Health, USA.

1 µg vitamin D_3_=40 international units (IU); 25(OH)D concentrations reported in ng/mL were converted to nmol/L (multiplying by 2.496)

*Sex missing for two participants randomised to intervention arm and subsequently excluded from analysis owing to lack of outcome data.

IPD integrity was confirmed by replication of primary analyses in published papers where applicable. The process of checking IPD identified three typographical errors in published reports. For the 2012 trial by Manaseki-Holland et al,^35^ the correct number of repeat episodes of chest radiography confirmed pneumonia was 134, rather than 138 as reported. For the trial by Dubnov-Raz et al,^36^ the number of patients randomised to the intervention arm was 27, rather than 28 as reported. For the trial by Laaksi et al,^37^ the proportion of men randomised to placebo who did not experience any acute respiratory tract infection was 30/84, rather than 30/80 as reported.

### Risk of bias within studies

Supplementary table S2 provides details of the risk of bias assessment. All but two trials were assessed as being at low risk of bias for all aspects assessed. Two trials were assessed as being at unclear risk of bias owing to high rates of loss to follow-up. In the trial by Dubnov-Raz et al,^36^ 52% of participants did not complete all symptom questionnaires. In the trial by Laaksi et al,^37^ 37% of randomised participants were lost to follow-up.

### Incidence of acute respiratory tract infection

#### Overall results

Table 2 presents the results of the one step IPD meta-analysis testing the effects of vitamin D on the proportion of all participants experiencing at least one acute respiratory tract infection, adjusting for age, sex, and study duration. Vitamin D supplementation resulted in a statistically significant reduction in the proportion of participants experiencing at least one acute respiratory tract infection (adjusted odds ratio 0.88, 95% confidence interval 0.81 to 0.96, P=0.003; P for heterogeneity <0.001; NNT=33, 95% confidence interval 20 to 101; 10 933 participants in 25 studies; see Cates plot, supplementary figure S1). Statistically significant protective effects of vitamin D were also seen for one step analyses of acute respiratory tract infection rate (adjusted incidence rate ratio 0.96, 95% confidence interval 0.92 to 0.997, P=0.04; P for heterogeneity <0.001; 10 703 participants in 25 studies) but not for analysis of time to first acute respiratory tract infection (adjusted hazard ratio 0.95, 95% confidence interval 0.89 to 1.01, P=0.09; P for heterogeneity <0.001; 9108 participants in 18 studies). Two step analyses also showed consistent effects for the proportion of participants experiencing at least one acute respiratory tract infection (adjusted odds ratio 0.80, 0.69 to 0.93, P=0.004; P for heterogeneity 0.001; 10 899 participants in 24 studies; fig 2), acute respiratory tract infection rate (adjusted incidence rate ratio 0.91, 0.84 to 0.98, P=0.018; P for heterogeneity <0.001; 10 703 participants in 25 studies), and time to first acute respiratory tract infection (adjusted hazard ratio 0.92, 0.85 to 1.00, P=0.051; P for heterogeneity 0.14; 9108 participants in 18 studies). This evidence was assessed as being of high quality (see supplementary table S3).

**Table 2 tbl2:** One step individual participant data meta-analysis, proportion of participants experiencing at least one acute respiratory tract infection (ARTI): overall and by subgroup

Variables	No of trials*	Proportion with ≥1 ARTI, control group (%)	Proportion with ≥1 ARTI, intervention group (%)	Adjusted odds ratio (95% CI)†	P value	P value for interaction
Overall	25	2204/5225 (42.2)	2303/5708 (40.3)	0.88 (0.81 to 0.96)	0.003	--
Baseline 25(OH)D (nmol/L):						
<25	14	137/249 (55.0)	117/289 (40.5)	0.58 (0.40 to 0.82)	0.002	0.01
≥25	19	1027/1639 (62.7)	1179/1995 (59.1)	0.89 (0.77 to 1.04)	0.15	
Dosing regimen type:						
Bolus dose ≥30 000 IU given	10	994/2786 (35.7)	1097/3014 (36.4)	0.97 (0.86 to 1.10)	0.67	0.05
Bolus dose not given	15	1210/2439 (49.6)	1206/2694 (44.8)	0.81 (0.72 to 0.91)	<0.001	
Daily dose equivalent (µg):						
<20	5	629/1321 (47.6)	619/1435 (43.1)	0.80 (0.68 to 0.94)	0.006	0.12
20-50	9	945/2796 (33.8)	1023/3077 (33.2)	0.90 (0.79 to 1.01)	0.08	
≥50	11	630/1108 (56.9)	661/1196 (55.3)	0.98 (0.81 to 1.18)	0.84	
Age (years):						
≤1	4	832/2744 (30.3)	854/2827 (30.2)	0.94 (0.83 to 1.06)	0.33	0.61
1.1-15.9	8	241/513 (47.0)	194/566 (34.3)	0.60 (0.46 to 0.77)	<0.001	
16-65	17	854/1459 (58.5)	885/1592 (55.6)	0.93 (0.79 to 1.10)	0.41	
>65	11	277/509 (54.4)	370/723 (51.2)	0.86 (0.67 to 1.09)	0.21	
Body mass index (kg/m^2^):						
<25	19	972/1943 (50.0)	956/2074 (46.1)	0.85 (0.74 to 0.97)	0.02	0.29
≥25	17	659/1039 (63.4)	754/1235 (61.1)	0.95 (0.79 to 1.14)	0.58	
Asthma:						
No	11	518/1008 (51.4)	520/1101 (47.2)	0.82 (0.68 to 0.99)	0.04	0.48
Yes	11	296/534 (55.4)	285/542 (52.6)	0.95 (0.73 to 1.25)	0.73	
COPD:						
No	7	477/763 (62.5)	493/791 (62.3)	1.00 (0.80 to 1.26)	0.98	0.38
Yes	6	122/230 (53.0)	120/238 (50.4)	0.84 (0.57 to 1.24)	0.38	
Influenza vaccination:						
No	10	255/373 (68.4)	253/407 (62.2)	0.74 (0.52 to 1.03)	0.08	0.51
Yes	10	564/779 (72.4)	577/826 (69.9)	0.86 (0.68 to 1.09)	0.22	

25(OH)D=25-hydroxyvitamin D; COPD=chronic obstructive pulmonary disease; 1 µg vitamin D_3_=40 international units (IU).

*Some trials did not contribute data to a given subgroup, either because individuals within that subgroup were not represented or because data relating to the potential effect modifier were not recorded; accordingly the number of trials represented varies between subgroups.

†Adjusted for age, sex, and study duration.

**Figure f2:**
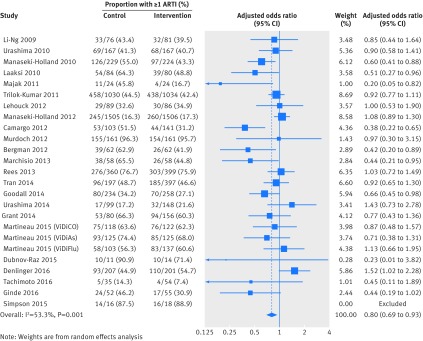
**Fig 2** Two step individual participant data meta-analysis: proportion of participants experiencing at least one acute respiratory tract infection (ARTI). Data from trial by Simpson et al were not included in this two step meta-analysis, as an estimate for the effect of the intervention in the study could not be obtained in the regression model owing to small sample size

#### Subgroup analyses

To explore reasons for heterogeneity, we conducted subgroup analyses to investigate whether effects of vitamin D supplementation on risk of acute respiratory tract infection differed according to baseline vitamin D status, dosing frequency, dose size, age, body mass index, the presence or absence of comorbidity (asthma or chronic obstructive pulmonary disease), and influenza vaccination status. Race or ethnicity was not investigated as a potential effect modifier, as data for this variable were missing for 3680/10 933 (34%) participants and power for subgroup analyses was limited by small numbers in many racial or ethnic subgroups that could not be meaningfully combined. Table 2 presents the results. Subgroup analysis revealed a strong protective effect of vitamin D supplementation among those with baseline circulating 25-hydroxyvitamin D levels less than 25 nmol/L (adjusted odds ratio 0.58, 0.40 to 0.82, NNT=8, 5 to 21; 538 participants in 14 studies; within subgroup P=0.002; see Cates plot, supplementary figure S1) and no statistically significant effect among those with baseline levels of 25 or more nmol/L (adjusted odds ratio 0.89, 0.77 to 1.04; 3634 participants in 19 studies; within subgroup P=0.15; P for interaction 0.01). This evidence was assessed as being of high quality (see supplementary table S3). An exploratory analysis testing the effects of vitamin D supplementation in those with baseline 25-hydroxyvitamin D concentrations in the ranges 25-49.9 nmol/L, 50-74.9 nmol/L, and 75 or more nmol/L did not reveal evidence of a statistically significant interaction (see supplementary table S4).

Meta-analysis of data from trials in which vitamin D was administered using a daily or weekly regimen without additional bolus doses revealed a protective effect against acute respiratory tract infection (adjusted odds ratio 0.81, 0.72 to 0.91, NNT=20, 13 to 43; 5133 participants in 15 studies; within subgroup P<0.001; see Cates plot, supplementary figure S1). No such protective effect was seen among participants in trials where at least one bolus dose of vitamin D was administered (adjusted odds ratio 0.97, 0.86 to 1.10; 5800 participants in 10 studies; within subgroup P=0.67; P for interaction 0.05). This evidence was assessed as being of high quality (see supplementary table S3). P values for interaction were more than 0.05 for all other potential effect modifiers investigated. For both of these subgroup analyses, broadly consistent effects were observed for event rate analysis (see supplementary table S5) and survival analysis (see supplementary table S6).

Having identified two potential factors that modified the influence of vitamin D supplementation on risk of acute respiratory tract infection (ie, baseline vitamin D status and dosing frequency), we then proceeded to investigate whether these factors were acting as independent effect modifiers, or whether they were confounded by each other or by another potential effect modifier, such as age. Dot plots revealed a trend towards lower median baseline serum 25-hydroxyvitamin D concentration and higher median age for studies employing bolus compared with daily or weekly dosing (see supplementary figures S2 and S3). To establish which of these potential effect modifiers was acting independently, we repeated the analysis to include treatment-covariate interaction terms for baseline vitamin D status, dosing frequency, and age. In this model, interaction terms for baseline vitamin D status and dosing frequency were statistically significant (P=0.01 and P=0.004, respectively), but the interaction term for age was not (P=0.20), consistent with the hypothesis that baseline vitamin D status and dosing frequency, but not age, independently modified the effect of vitamin D supplementation on risk of acute respiratory tract infection.

We then proceeded to stratify the subgroup analysis presented in table 2 according to dosing frequency, to provide a “cleaner” look at the results of subgroup analyses under the assumption that use of bolus doses was ineffective. Table 3 presents the results: these reveal that daily or weekly vitamin D treatment was associated with an even greater degree of protection against acute respiratory tract infection among participants with baseline circulating 25-hydroxyvitamin D concentrations less than 25 nmol/L than in the unstratified analysis (adjusted odds ratio 0.30, 0.17 to 0.53; NNT=4, 3 to 7; 234 participants in six studies; within subgroup P<0.001; see Cates plot, supplementary figure S4). Moreover, use of daily or weekly vitamin D also protected against acute respiratory tract infection among participants with higher baseline 25-hydroxyvitamin D concentrations (adjusted odds ratio 0.75, 0.60 to 0.95; NNT=15, 9 to 86; 1603 participants in six studies; within subgroup P=0.02; see Cates plot, supplementary figure S4). The P value for interaction for this subgroup analysis was 0.006, indicating that protective effects of daily or weekly vitamin D supplementation were statistically significantly greater in the subgroup of participants with profound vitamin D deficiency. No other statistically significant interaction was seen; notably, bolus dose vitamin D supplementation did not offer any protection against acute respiratory tract infection even when administered to those with circulating 25-hydroxyvitamin D concentrations less than 25 nmol/L (adjusted odds ratio 0.82, 0.51 to 1.33; 304 participants in eight studies; within subgroup P=0.43).

**Table 3 tbl3:** One step individual participant data meta-analysis, proportion of participants experiencing at least one acute respiratory tract infection (ARTI): overall and by subgroup, stratified by dosing frequency

Variables	Bolus dosing							Daily or weekly dosing					
	No of trials*	Proportion with ≥1 ARTI, control group (%)	Proportion with ≥1 ARTI, intervention group (%)	Adjusted odds ratio (95% CI)†	P value	P value for interaction		No of trials*	Proportion with ≥1 ARTI, control group (%)	Proportion with ≥1 ARTI, intervention group (%)	Adjusted odds ratio (95% CI)†	P value	P value for interaction
Overall	10	994/2786 (35.7)	1097/3014 (36.4)	0.97 (0.86 to 1.10)	0.67	--		15	1210/2439 (49.6)	1206/2694 (44.8)	0.81 (0.72 to 0.91)	0.001	--
Baseline 25(OH)D (nmol/L):													
<25	8	73/142 (51.4)	77/162 (47.5)	0.82 (0.51 to 1.33)	0.43	0.42		6	64/107 (59.8)	40/127 (31.5)	0.30 (0.17 to 0.53)	<0.001	0.006
≥25	8	550/910 (60.4)	663/1121 (59.1)	1.02 (0.83 to 1.24)	0.87			11	477/729 (65.4)	516/874 (59.0)	0.75 (0.60 to 0.95)	0.02	
Daily dose equivalent (µg):													
<20	.	.	.	.	.	0.56		5	629/1321 (47.6)	619/1435 (43.1)	0.80 (0.68 to 0.94)	0.006	0.82
20-50	3	467/1931 (24.2)	542/2127 (25.5)	0.95 (0.81 to 1.10)	0.50			6	478/865 (55.3)	481/950 (50.6)	0.81 (0.66 to 1.01)	0.06	
≥50	7	527/855 (61.6)	555/887 (62.6)	1.03 (0.83 to 1.28)	0.81			4	103/253 (40.7)	106/309 (34.3)	0.85 (0.58 to 1.24)	0.39	
Age (years):													
≤1	2	321/1634 (19.6)	322/1637 (19.7)	0.99 (0.83 to 1.19)	0.93	0.72		2	511/1110 (46.0)	532/1190 (44.7)	0.91 (0.77 to 1.08)	0.30	0.37
1.1-15.9	1	50/100 (50.0)	35/93 (37.6)	0.62 (0.35 to 1.11)	0.11			7	191/413 (46.2)	159/473 (33.6)	0.59 (0.45 to 0.79)	<0.001	
16-65	8	432/678 (63.7)	466/716 (65.1)	1.15 (0.90 to 1.48)	0.27			9	422/781 (54.0)	419/876 (47.8)	0.79 (0.63 to 0.99)	0.04	
>65	8	191/374 (51.1)	274/568 (48.2)	0.85 (0.65 to 1.12)	0.25			3	86/135 (63.7)	96/155 (61.9)	0.88 (0.52 to 1.52)	0.66	
Body mass index (kg/m^2^):													
<25	8	215/372 (57.8)	231/417 (55.4)	1.01 (0.72 to 1.40)	0.97	0.70		11	757/1571 (48.2)	725/1657 (43.8)	0.82 (0.71 to 0.95)	0.009	>0.99
≥25	8	406/677 (60.0)	509/867 (58.7)	1.00 (0.80 to 1.25)	0.98			9	253/358 (70.7)	245/367 (66.8)	0.83 (0.59 to 1.17)	0.30	
Asthma:													
No	5	303/484 (62.6)	323/523 (61.8)	0.95 (0.71 to 1.28)	0.75	0.40		6	215/524 (41.0)	197/578 (34.1)	0.74 (0.58 to 0.95)	0.02	0.40
Yes	4	224/371 (60.4)	232/364 (63.7)	1.18 (0.85 to 1.65)	0.32			7	72/163 (44.2)	53/178 (29.8)	0.60 (0.37 to 0.98)	0.04	
COPD:													
No	5	410/632 (64.9)	436/656 (66.5)	--‡	--‡	--‡		2	67/131 (51.1)	57/135 (42.2)	--‡	--‡	--‡
Yes	4	117/223 (52.5)	119/231 (51.5)	--‡	--‡	--‡		2	5/7 (71.4)	1/7 (14.3)	--‡	--‡	--‡
Influenza vaccination													
No	5	119/163 (73.0)	121/178 (68.0)	--‡	--‡	--‡		5	136/210 (64.8)	132/229 (57.6)	--‡	--‡	--‡
Yes	5	286/396 (72.2)	294/421 (69.8)	.	.	.		5	278/383 (72.6)	283/405 (69.9)			

25(OH)D=25-hydroxyvitamin D; COPD=chronic obstructive pulmonary disease; 1 µg vitamin D_3_=40 international units (IU).

*Some trials did not contribute data to a given subgroup, either because individuals within that subgroup were not represented or because data relating to the potential effect modifier were not recorded; accordingly the number of trials represented varies between subgroups.

†Adjusted for age, sex, and study duration.

‡Values could not be estimated as models did not converge.

### Secondary outcomes

#### Efficacy

Table 4 presents the results of the one step IPD meta-analysis of secondary outcomes. When all studies were analysed together, no statistically significant effect of vitamin D was seen on the proportion of participants with at least one upper respiratory tract infection, lower respiratory tract infection, hospital admission or emergency department attendance for acute respiratory tract infection, course of antimicrobials for acute respiratory tract infection, or absence from work or school due to acute respiratory tract infection. However, when this analysis was stratified by dosing frequency, a borderline statistically significant protective effect of daily or weekly vitamin D supplementation against upper respiratory tract infection was seen (adjusted odds ratio 0.88, 0.78 to 1.00; 4483 participants in 11 studies, P=0.05; table 5).

**Table 4 tbl4:** One step individual participant data meta-analysis of secondary outcomes

Outcomes	No of trials	Proportion with ≥1 event		Adjusted odds ratio (95% CI)*	P value
		Control group (%)	Intervention group (%)		
Upper respiratory tract infection	19	1656/3286 (50.4)	1807/3733 (48.4)	0.93 (0.83 to 1.03)	0.15
Lower respiratory tract infection	9	542/3285 (16.5)	561/3413 (16.4)	0.96 (0.83 to 1.10)	0.52
Hospital admission or emergency department attendance due to ARTI	11	47/3886 (1.2)	40/3986 (1.0)	0.83 (0.54 to 1.27)	0.39
Use of antimicrobials for treatment of ARTI	9	397/983 (40.4)	413/1121 (36.8)	0.84 (0.69 to 1.03)	0.10
Work or school absence due to ARTI	7	321/632 (50.8)	319/684 (46.6)	0.87 (0.69 to 1.09)	0.22
Serious adverse event of any cause	25	216/5371 (4.0)	221/5853 (3.8)	0.98 (0.80 to 1.20)	0.83
Death due to ARTI or respiratory failure	25	7/5330 (0.1)	6/5802 (0.1)	0.70 (0.23 to 2.20)	0.55
Death due to any infection	25	15/5338 (0.3)	16/5812 (0.3)	0.95 (0.46 to 1.99)	0.90
Death due to any cause	25	48/5371 (0.9)	56/5853 (1.0)	1.39 (0.85 to 2.27)	0.18
Hypercalcaemia	14	9/1739 (0.5)	12/2111 (0.6)	--†	--†
Renal stones	14	4/1707 (0.2)	2/2134 (0.1)	--†	--†

ARTI=acute respiratory tract infection.

*Adjusted for age, sex, and study duration.

†values could not be estimated as models did not converge.

**Table 5 tbl5:** One step individual participant data meta-analysis of secondary outcomes, stratified by dosing frequency

Outcomes	Bolus dosing						Daily or weekly dosing				
	No of trials	Proportion with ≥1 event, control group (%)	Proportion with ≥1 event, intervention group (%)	Adjusted odds ratio (95% CI)*	P value		No of trials	Proportion with ≥1 event, control group (%)	Proportion with ≥1 event, intervention group (%)	Adjusted odds ratio (95% CI)*	P value
Upper respiratory tract infection	8	606/1052 (57.6)	730/1284 (56.9)	1.03 (0.86 to 1.24)	0.72		11	1050/2234 (47.0)	1077/2449 (44.0)	0.88 (0.78 to 1.00)	0.05
Lower respiratory tract infection	4	424/1889 (22.4)	427/1922 (22.2)	0.96 (0.82 to 1.13)	0.60		5	118/1396 (8.5)	134/1491 (9.0)	0.98 (0.75 to 1.28)	0.88
Use of antimicrobials for treatment of ARTI	4	201/348 (57.8)	203/367 (55.3)	0.79 (0.56 to 1.10)	0.16		5	196/635 (30.9)	210/754 (27.9)	0.87 (0.67 to 1.13)	0.31
Work or school absence due to ARTI	4	219/409 (53.5)	196/411 (47.7)	0.78 (0.59 to 1.04)	0.10		3	102/223 (45.7)	123/273 (45.1)	1.03 (0.71 to 1.48)	0.88
Serious adverse event of any cause	10	107/2822 (3.8)	115/3070 (3.7)	1.00 (0.74 to 1.35)	0.99		15	109/2549 (4.3)	106/2783 (3.8)	0.97 (0.73 to 1.30)	0.86
Death due to any cause	10	29/2822 (1.0)	35/3070 (1.1)	1.29 (0.71 to 2.35)	0.40		15	19/2549 (0.7)	21/2783 (0.8)	--†	--†
Death due to ARTI or respiratory failure	10	4/2797 (0.1)	3/3038 (0.1)	0.61 (0.12 to 3.02)	0.54		15	3/2533 (0.1)	3/2765 (0.1)	--†	--†
Death due to any infection	10	8/2801 (0.3)	5/3040 (0.2)	0.55 (0.17 to 1.80)	0.32		15	7/2537 (0.3)	11/2773 (0.4)	--†	--†
Hospital admission or emergency department attendance due to ARTI	6	4/2081 (0.2)	6/2124 (0.3)	--†	--†		5	43/1805 (2.4)	34/1862 (1.8)	--†	--†
Hypercalcaemia	8	8/1062 (0.8)	11/1303 (0.8)	--†	--†		6	1/677 (0.1)	1/808 (0.1)	--†	--†
Renal stones	6	0/764 (0.0)	1/1011 (0.1)	--†	--†		8	4/943 (0.4)	1/1123 (0.1)	--†	--†

ARTI=acute respiratory tract infection.

*Adjusted for age, sex, and study duration.

†Values could not be estimated as model did not converge.

#### Safety

Use of vitamin D did not influence risk of serious adverse events of any cause (adjusted odds ratio 0.98, 0.80 to 1.20; 11 224 participants in 25 studies) or death due to any cause (1.39, 0.85 to 2.27; 11 224 participants in 25 studies) (table 4). Instances of potential adverse reactions to vitamin D were rare. Hypercalcaemia was detected in 21/3850 (0.5%) and renal stones were diagnosed in 6/3841 (0.2%); both events were evenly represented between intervention and control arms (table 4). Stratification of this analysis by dosing frequency did not reveal any statistically significant increase in risk of adverse events with either bolus dosing or daily or weekly supplementation (table 5).

### Risk of bias across studies

A funnel plot for the proportion of participants experiencing at least one acute respiratory tract infection showed a degree of asymmetry, raising the possibility that small trials showing adverse effects of vitamin D might not have been included in the meta-analysis (see supplementary figure S5).

### Responder analyses

Supplementary table S7 presents the results of responder analyses. Among participants randomised to the intervention arm of included studies for whom end study data on 25-hydroxyvitamin D were available, no difference in risk of acute respiratory tract infection was observed between those who attained a serum concentration of 75 or more nmol/L compared with those who did not.

### Sensitivity analyses

IPD meta-analysis of the proportion of participants experiencing at least one acute respiratory tract infection, excluding two trials assessed as being at unclear risk of bias,^36^ ^37^ revealed protective effects of vitamin D supplementation consistent with the main analysis (adjusted odds ratio 0.82, 0.70 to 0.95, 10 744 participants, P=0.01). Sensitivity analysis for the same outcome, restricted to the 14 trials that investigated acute respiratory tract infection as the primary or coprimary outcome, also revealed protective effects of vitamin D supplementation consistent with the main analysis (0.82, 0.68 to 1.00, 5739 participants, P=0.05).

## Discussion

In this individual participant data (IPD) meta-analysis of randomised controlled trials, vitamin D supplementation reduced the risk of experiencing at least one acute respiratory tract infection. Subgroup analysis revealed that daily or weekly vitamin D supplementation without additional bolus doses protected against acute respiratory tract infection, whereas regimens containing large bolus doses did not. Among those receiving daily or weekly vitamin D, protective effects were strongest in those with profound vitamin D deficiency at baseline, although those with higher baseline 25-hydroxyvitamin D concentrations also experienced benefit. This evidence was assessed as being of high quality, using the GRADE criteria.^34^ Since baseline vitamin D status and use of bolus doses varied considerably between studies, our results suggest that the high degree of heterogeneity between trials may be at least partly attributable to these factors. Use of vitamin D was safe: potential adverse reactions were rare, and the risk of such events was the same between participants randomised to intervention and control arms.

Why might use of bolus dose vitamin D be ineffective for prevention of acute respiratory tract infection? One explanation relates to the potentially adverse effects of wide fluctuations in circulating 25-hydroxyvitamin D concentrations, which are seen after use of bolus doses but not with daily or weekly supplementation. Vieth has proposed that high circulating concentrations after bolus dosing may chronically dysregulate activity of enzymes responsible for synthesis and degradation of the active vitamin D metabolite 1,25-dihydroxyvitamin D, resulting in decreased concentrations of this metabolite in extra-renal tissues.^38^ Such an effect could attenuate the ability of 25-hydroxyvitamin D to support protective immune responses to respiratory pathogens. Increased efficacy of vitamin D supplementation in those with lower baseline vitamin D status is more readily explicable, based on the principle that people who are the most deficient in a micronutrient will be the most likely to respond to its replacement.

### Strengths and limitations of this study

Our study has several strengths. We obtained IPD for all 25 trials identified by our search; the proportion of randomised participants with missing outcome data was small (3.4%); participants with diverse characteristics in multiple settings were represented; and 25-hydroxyvitamin D levels were measured using validated assays in laboratories that participated in external quality assessment schemes. Our findings therefore have a high degree of internal and external validity. Moreover, the subgroup effects we report fulfil published “credibility criteria” relating to study design, analysis, and context.^39^ Specifically, the relevant effect modifiers were specified a priori and measured at baseline, P values for interaction remained significant after adjustment for potential confounders, and subgroup effects were consistent when analysed as proportions and event rates. Survival analysis revealed consistent trends that did not attain statistical significance, possibly owing to lack of power (fewer studies contributed data to survival analyses than to analyses of proportions and event rates). The concepts that vitamin D supplementation may be more effective when given to those with lower baseline 25-hydroxyvitamin D levels and less effective when bolus doses are administered, are also biologically plausible. A recent Cochrane review of randomised controlled trials reporting that vitamin D supplementation reduces the risk of severe asthma exacerbations, which are commonly precipitated by viral upper respiratory tract infections, adds further weight to the case for biological plausibility.^40^ Although the results are consistent with the hypothesis that baseline vitamin D status and dosing regimen independently modify the effects of vitamin D supplementation, we cannot exclude the possible influence of other effect modifiers linked to these two factors. The risk of residual confounding by other effect modifiers is increased for analyses where relatively few trials are represented within a subgroup—for example, where subgroup analyses were stratified by dosing regimen. We therefore suggest caution when interpreting the results in table 3.

Our study has some limitations. One explanation for the degree of asymmetry seen in the funnel plot is that some small trials showing adverse effects of vitamin D might have escaped our attention. With regard to the potential for missing data, we made strenuous efforts to identify published and (at the time) unpublished data, as illustrated by the fact that our meta-analysis includes data from 25 studies—10 more than the largest aggregate data meta-analysis on the topic.^13^ However, if one or two small trials showing large adverse effects of vitamin D were to emerge, we do not anticipate that they would greatly alter the results of the one step IPD meta-analysis, since any negative signal from a modest number of additional participants would likely be diluted by the robust protective signal generated from analysis of data from nearly 11 000 participants. A second limitation is that our power to detect effects of vitamin D supplementation was limited for some subgroups (eg, individuals with baseline 25-hydroxyvitamin D concentrations <25 nmol/L receiving bolus dosing regimens) and for some secondary outcomes (eg, incidence of lower respiratory tract infection). Null and borderline statistically significant results for analyses of these outcomes may have arisen as a consequence of type 2 error. Additional randomised controlled trials investigating the effects of vitamin D on risk of acute respiratory tract infection are ongoing, and inclusion of data from these studies in future meta-analyses has the potential to increase statistical power to test for subgroup effects. However, all three of the largest such studies (NCT01169259, ACTRN12611000402943, and ACTRN12613000743763) are being conducted in populations where profound vitamin D deficiency is rare, and two are using intermittent bolus dosing regimens: the results are therefore unlikely to alter our finding of benefit in people who are very deficient in vitamin D or in those receiving daily or weekly supplementation. A third potential limitation is that data relating to adherence to study drugs were not available for all participants. However, inclusion of non-adherent participants would bias results of our intention to treat analysis towards the null: thus we conclude that effects of vitamin D in those who are fully adherent to supplementation will be no less than those reported for the study population overall. Finally, we caution that study definitions of acute respiratory tract infection were diverse, and virological, microbiological, or radiological confirmation was obtained for the minority of events. Acute respiratory tract infection is often a clinical diagnosis in practice, however, and since all studies were double blind and placebo controlled, differences in incidence of events between study arms cannot be attributed to observation bias.

### Conclusions and policy implications

Our study reports a major new indication for vitamin D supplementation: the prevention of acute respiratory tract infection. We also show that people who are very deficient in vitamin D and those receiving daily or weekly supplementation without additional bolus doses experienced particular benefit. Our results add to the body of evidence supporting the introduction of public health measures such as food fortification to improve vitamin D status, particularly in settings where profound vitamin D deficiency is common.

**What is already known on this topic**

Randomised controlled trials of vitamin D supplementation for the prevention of acute respiratory tract infection have yielded conflicting results
Individual participant data (IPD) meta-analysis has the potential to identify factors that may explain this heterogeneity, but this has not previously been performed

**What this study adds**

Meta-analysis of IPD from 10 933 participants in 25 randomised controlled trials showed an overall protective effect of vitamin D supplementation against acute respiratory tract infection (number needed to treat (NNT)=33)
Benefit was greater in those receiving daily or weekly vitamin D without additional bolus doses (NNT=20), and the protective effects against acute respiratory tract infection in this group were strongest in those with profound vitamin D deficiency at baseline (NNT=4)
These findings support the introduction of public health measures such as food fortification to improve vitamin D status, particularly in settings where profound vitamin D deficiency is common

## Web extra

Extra material supplied by authors

## Notes

We thank the participants in the primary randomised controlled trials; the teams who conducted the trials; our patient and public involvement representatives Charanjit Patel and Jane Gallagher for comments on study design and drafts of this manuscript; and Khalid S Khan, Queen Mary University of London, for valuable advice and helpful discussions.

Contributors: ARM led the funding application, with input from RLH, CJG, and CAC who were co-applicants. ARM, DAJ, and CAC assessed eligibility of studies for inclusion. ARM, JFA, PB, GD-R, SE, DG, AAG, ECG, CCG, WJ, IL, SM-H, DM, DRM, RN, JRR, SS, IS, GTK, MU, and CAC were all directly involved in the acquisition of data for the work. RLH, LG, ARM, and DAJ designed the statistical analyses in consultation with authors contributing individual patient data. Statistical analyses were done by LG, RLH, and DAJ. ARM wrote the first draft of the report. He is the guarantor. All authors revised it critically for important intellectual content, gave final approval of the version to be published, and agreed to be accountable for all aspects of the work in ensuring that questions related to the accuracy or integrity of any part of the work were appropriately investigated and resolved.

Ethical approval: Not required.

Data sharing: A partial dataset, incorporating patient level data from trials for which the relevant permissions for data sharing have been obtained, is available from the corresponding author at a.martineau@qmul.ac.uk.

Transparency: The manuscript’s guarantor (ARM) affirms that the manuscript is an honest, accurate, and transparent account of the study being reported and that no important aspects of the study have been omitted. All analyses were prespecified in the study protocol, other than those presented in tables 3 and 5, which were conducted in response to a reviewer’s request.
