# Supplementary material for: Vitamin D supplementation to prevent acute respiratory tract infections: systematic review and meta-analysis of individual participant data
Source: BMJ. 2017 Feb 15;356:i6583. doi: 10.1136/bmj.i6583 (PMC5310969; doi:10.1136/bmj.i6583)
Supplement: Supplementary file 1 — Supplementary information: Supplementary material [file mara034434.ww1_default.pdf]

Vitamin D supplementation to prevent acute respiratory tract infections: systematic review and meta-analysis of individual participant data

Supplementary Material

Adrian R Martineau[1,2†]
David A Jolliffe[1]
Richard L Hooper[1]
Lauren Greenberg[1]
John F Aloia[3]
Peter Bergman[4]
Gal Dubnov-Raz[5]
Susanna Esposito[6]
Davaasambuu Ganmaa[7]
Adit A Ginde[8]
Emma C Goodall[9]
Cameron C Grant[10]
Christopher J Griffiths[1,2,11]
Wim Janssens[12]
Ilkka Laaksi[13]
Semira Manaseki-Holland[14]
David Mauger[15]
David R Murdoch[16]
Rachel Neale[17]
Judy R Rees[18]
Steve Simpson Jr[19]
Iwona Stelmach[20]
Geeta Trilok Kumar[21]
Mitsuyoshi Urashima[22]
Carlos A Camargo Jr[23]

[1]Centre for Primary Care and Public Health, Barts and The London School of Medicine and Dentistry, Queen Mary University of London, London, UK
[2]Asthma UK Centre for Applied Research, Blizard Institute, Queen Mary University of London, London, UK
[3]Bone Mineral Research Center, Winthrop University Hospital, Mineola, NY, USA
[4]Department of Laboratory Medicine, Karolinska Institutet, Stockholm, Sweden
[5]Dept. of Exercise, Lifestyle and Nutrition Clinic, Edmond and Lily Safra Children's Hospital, Tel Hashomer, Israel
[6] Pediatric Highly Intensive Care Unit, Fondazione IRCCS Ca' Granda Ospedale Maggiore Policlinico, Università degli Studi di Milano, Milan, Italy
[7]Department of Nutrition, Harvard School of Public Health, Boston, MA, USA
[8]Department of Emergency Medicine, University of Colorado School of Medicine, Aurora, CO, USA
[9]Department of Clinical Epidemiology and Biostatistics, McMaster University, Hamilton, Ontario, Canada
[10]Department of Paediatrics: Child & Youth Health, Faculty of Medical and Health Sciences, University of Auckland, Auckland, New Zealand
[11]MRC and Asthma UK Centre in Allergic Mechanisms of Asthma, Blizard Institute, Queen Mary University of London, London, UK
[12]Universitair ziekenhuis Leuven, Leuven, Belgium
[13]Tampere School of Public Health, University of Tampere, Tampere, Finland 33014
[14]Department of Public Health, Epidemiology and Biostatistics, Institute of Applied Health Sciences, College of Medical and Dental Sciences, University of Birmingham, Birmingham, UK
[15]Department of Statistics, The Pennsylvania State University, Hershey, PA, USA
[16]Department of Pathology, University of Otago, Christchurch, New Zealand
[17]QIMR Berghofer Medical Research Institute, Queensland, Australia
[18] Department of Epidemiology, Geisel School of Medicine at Dartmouth, Lebanon, NH, USA
[19]Menzies Institute for Medical Research, University of Tasmania, Hobart, Australia
[20]Department of Pediatrics and Allergy, Medical University of Lodz, Lodz, Poland
[21]Institute of Home Economics, University of Delhi, New Delhi, India
[22]Division of Molecular Epidemiology, Jikei University School of Medicine, Tokyo, Japan
[23]Department of Emergency Medicine, Massachusetts General Hospital, Harvard Medical School, Boston, MA, USA

† To whom correspondence should be addressed at Centre for Primary Care and Public Health, Blizard Institute, Barts and The London School of Medicine and Dentistry, Queen Mary University of London, 58 Turner St, London E1 2AB, UK
Tel: +44 207 882 2551 │Fax: +44 207 882 2552 │Email: a.martineau@qmul.ac.uk

Search Strategies

A. Medline

Cochrane Highly Sensitive Search Strategy for identifying randomised controlled trials

#1. randomized controlled trial [pt] OR controlled clinical trial [pt] OR randomized [tiab] OR placebo [tiab] OR drug therapy [sh] OR randomly [tiab] OR trial [tiab] OR groups [tiab]

#2. animals [mh] NOT humans [mh]

#3. #1 NOT #2

Terms specific to vitamin D

#4. Vitamin D OR vitamin D2 OR vitamin D3 OR cholecalciferol OR ergocalciferol OR alphacalcidol OR alfacalcidol OR calcitriol OR paricalcitol OR doxerocalciferol

Terms specific to acute respiratory tract infection

#5. Acute Respiratory Infection OR Upper Respiratory Infection OR Lower Respiratory Infection OR Respiratory Tract Infection OR Common Cold OR Sinusitis OR Pharyngitis OR Laryngitis OR Laryngotracheobronchitis OR Tonsillitis OR peritonsillar abscess OR Croup OR Epiglottitis OR supraglottitis OR Otitis Media OR Pneumonia OR Bronchopneumonia OR Bronchitis OR Pleurisy OR Pleuritis

Combination of terms to identify randomised controlled trials of vitamin D for the prevention of acute respiratory infection

#3 AND #4 AND #5

B. EMBASE

Terms for identifying randomised controlled trials

#1 ‘randomized controlled trial’/exp OR ‘single blind procedure’/exp OR ‘double blind procedure’/exp OR ’crossover procedure’/exp
#2 random*:ab,ti OR placebo*:ab,ti OR crossover*:ab,ti OR ’cross over’:ab,ti OR allocat*:ab,ti OR ((singl* OR doubl*) NEXT/1 blind*):ab,ti OR trial:ti
#3. #1 OR #2

Terms specific to vitamin D

#4. ***vitamin AND d OR vitamin AND d2 OR vitamin AND d3 OR cholecalciferol OR ergocalciferol OR alphacalcidol OR alfacalcidol OR calcitriol OR paricalcitol OR doxerocalciferol***

Terms specific to acute respiratory tract infection

#5. acute AND respiratory AND infection OR upper AND respiratory AND infection OR lower AND respiratory AND infection OR respiratory AND tract AND infection OR common AND cold OR sinusitis OR pharyngitis OR laryngitis OR laryngotracheobronchitis OR tonsillitis OR peritonsillar AND abscess OR croup OR epiglottitis OR supraglottitis OR otitis AND media OR pneumonia OR bronchopneumonia OR bronchitis OR pleurisy OR pleuritis

Combination of terms to identify randomised controlled trials of vitamin D for the prevention of acute respiratory tract infection

#3 AND #4 AND #5

C. Cochrane Central

Terms specific to vitamin D

#1. Vitamin D OR vitamin D2 OR vitamin D3 OR cholecalciferol OR ergocalciferol OR alphacalcidol OR alfacalcidol OR calcitriol OR paricalcitol OR doxerocalciferol

Terms specific to acute respiratory tract infection

#2. Acute Respiratory Infection OR Upper Respiratory Infection OR Lower Respiratory Infection OR Respiratory Tract Infection OR Common Cold OR Sinusitis OR Pharyngitis OR Laryngitis OR Laryngotracheobronchitis OR Tonsillitis OR peritonsillar abscess OR Croup OR Epiglottitis OR supraglottitis OR Otitis Media OR Pneumonia OR Bronchopneumonia OR Bronchitis OR Pleurisy OR Pleuritis

Combination of terms to identify randomised controlled trials of vitamin D for the prevention of acute respiratory tract infection

#1 AND #2

D. Web of Science

TS =(Vitamin D OR vitamin D2 OR vitamin D3 OR cholecalciferol OR ergocalciferol OR alphacalcidol OR alfacalcidol OR calcitriol OR paricalcitol OR doxerocalciferol) AND TS =(Acute Respiratory Infection OR Upper Respiratory Infection OR Lower Respiratory Infection OR Respiratory Tract Infection OR Common Cold OR Sinusitis OR Pharyngitis OR Laryngitis OR Laryngotracheobronchitis OR Tonsillitis OR peritonsillar abscess OR Croup OR Epiglottitis OR supraglottitis OR Otitis Media OR Pneumonia OR Bronchopneumonia OR Bronchitis OR Pleurisy OR Pleuritis) AND TS =(placebo* or random* or clinical trial* or double blind* or single blind* or rct)

E. ClinicalTrials.gov and ISRCTN Registry

Vitamin D AND respiratory AND infection

Sources of support for participating trials

The trial by Ginde and colleagues was supported by NIH/NIA grant K23AG040708, NIH/NCATS Colorado CTSA Grant UL1TR001082, and the American Geriatrics Society Jahnigen Career Development Scholars Award

The trial by Goodall and colleagues was supported in part by the Canadian Institutes of Health Research [OPP 86940] and with in-kind support from Copan Italia, Bresica Italy.

The trial by Grant and colleagues was supported by the Health Research Council of New Zealand, Grant Number 09/215R.

The trials by Manaseki-Holland and colleagues were supported by New Zealand Aid (ref: GRA/470/2) and The Wellcome Trust (ref: 082476).

The trials by Martineau and colleagues were supported by the National Institute for Health Research under its Programme Grants for Applied Research Programme (Reference Number RP-PG-0407-10398).

The trial by Simpson and colleagues was supported by the Royal Hobart Hospital Research Foundation.

The trial by Trilok Kumar and colleagues was supported by the Department of Biotechnology, Government of India (ref BT/PR-PR7489/PID/20/285/2006), Nutrition Third World and Sight and Life.

The trial by Rees and colleagues was supported by the National Cancer Institute at the National Institutes of Health (grant numbers CA098286 and CA098286-S).

The trial by Tran and colleagues was supported by the National Health and Medical Research Council of Australia, grant 613655.

Results

Table S1: Participants' baseline characteristics by study arm

	Control group (n=5,225)	Intervention group (n=5,708)
**Sex**		
Male, N (%)	2,564 (49.1)	2,871 (50.3)
Female, N (%)	2,659 (50.9)	2,836 (49.7)
**Age, years**		
≤1	2744 (52.5)	2827 (49.5)
1.1-15.9	513 (9.8)	566 (27.9)
16-65	1459 (27.9)	1592 (27.9)
>65	509 (9.7)	723 (12.7)
**25(OH)D, nmol/L**		
<25	249 (4.8)	289 (5.1)
≥25	1639 (31.4)	1995 (35.0)
Not recorded	3337 (63.9)	3424 (60.0)
**Body mass index, kg/m^2**		
<25	1943 (37.2)	2074 (36.3)
≥25	1039 (19.9)	1235 (21.6)
Not recorded	2243 (42.9)	2399 (42.0)
**Co-morbidity: Asthma**		
No	1008 (19.3)	1101 (19.3)
Yes	534 (10.2)	542 (9.5)
Not recorded	3683 (70.5)	4065 (71.2)
**Co-morbidity: COPD**		
No	763 (14.6)	791 (13.9)
Yes	230 (4.4)	238 (4.2)
Not recorded	4232 (81.0)	4679 (82.0)
**Influenza vaccination**		
No	373 (7.1)	407 (7.1)
Yes	779 (14.9)	826 (14.5)
Not recorded	4073 (78.0)	4475 (78.4)

Abbreviations: 25(OH)D, 25-hydroxyvitamin D; COPD, Chronic Obstructive Pulmonary Disease

Table S2: Risk of Bias Assessment

	Sequence generation	Allocation concealment	Blinding of participants and personnel	Blinding of outcome assessment	Incomplete outcome data	Selective reporting	Other bias
Li-Ng 2009[1]	✓	✓	✓	✓	✓	✓	✓
Urashima 2010[2]	✓	✓	✓	✓	✓	✓	✓
Manaseki-Holland 2010[3]	✓	✓	✓	✓	✓	✓	✓
Laaksi 2010[4]	✓	✓	✓	✓	?	✓	✓
Majak 2011[5]	✓	✓	✓	✓	✓	✓	✓
Trilok-Kumar 2011[6]	✓	✓	✓	✓	✓	✓	✓
Lehouck 2012[7]	✓	✓	✓	✓	✓	✓	✓
Manaseki-Holland 2012[8]	✓	✓	✓	✓	✓	✓	✓
Camargo 2012[9]	✓	✓	✓	✓	✓	✓	✓
Murdoch 2012[10]	✓	✓	✓	✓	✓	✓	✓
Bergman 2012[11]	✓	✓	✓	✓	✓	✓	✓
Marchisio 2013[12]	✓	✓	✓	✓	✓	✓	✓
Rees 2013[13]	✓	✓	✓	✓	✓	✓	✓
Tran 2014[14]	✓	✓	✓	✓	✓	✓	✓
Goodall 2014[15]	✓	✓	✓	✓	✓	✓	✓
Urashima 2014[16]	✓	✓	✓	✓	✓	✓	✓
Grant 2014[17]	✓	✓	✓	✓	✓	✓	✓
Martineau 2015a[18] [ViDiCO]	✓	✓	✓	✓	✓	✓	✓
Martineau 2015b[19] [ViDiAs]	✓	✓	✓	✓	✓	✓	✓
Martineau 2015c[20] [ViDiFlu]	✓	✓	✓	✓	✓	✓	✓
Simpson 2015[21]	✓	✓	✓	✓	✓	✓	✓
Dubnov-Raz 2015[22]	✓	✓	✓	✓	?	✓	✓
Denlinger 2016[23]	✓	✓	✓	✓	✓	✓	✓
Tachimoto 2016[24]	✓	✓	✓	✓	✓	✓	✓
Ginde 2016[25]	✓	✓	✓	✓	✓	✓	✓

✓ = low risk of bias; ? = unclear risk of bias;

Table S3: Summary of Findings Table

Vitamin D_3 compared to placebo for prevention of acute respiratory tract infection (ARTI)

Population: children and adults of any age, sex or ethnic origin, with or without co-morbidity
Setting: fourteen countries on four continents (Asia, Australasia, Europe, North America)
Intervention: oral vitamin D_3 (cholecalciferol) supplementation
Comparison: oral placebo

Outcomes	Anticipated absolute effects* (95% CI)		Relative effect (95% CI)	№ of participants (studies)	Quality of the evidence (GRADE)
	**Risk with placebo**	**Risk with Vitamin D**			
Proportion with at least one ARTI, all participants	422 per 1,000	391 per 1,000 (371 to 412)	**OR 0.88** (0.81 to 0.96)	10933 (25 RCTs)	⨁⨁⨁⨁ HIGH
Proportion with at least one ARTI, baseline 25(OH)D <25 nmol/L	408 per 1,000	299 per 1,000 (237 to 364)	**OR 0.62** (0.45 to 0.83)	871 (15 RCTs)	⨁⨁⨁⨁ HIGH
Proportion with at least one ARTI, daily or weekly vitamin D (no boluses)	496 per 1,000	444 per 1,000 (415 to 473)	**OR 0.81** (0.72 to 0.91)	5133 (15 RCTs)	⨁⨁⨁⨁ HIGH
Proportion with serious adverse event, any cause	40 per 1,000	39 per 1,000 (32 to 48)	**OR 0.98** (0.80 to 1.20)	11224 (25 RCTs)	⨁⨁⨁⨁ HIGH

***The risk in the intervention group** (and its 95% confidence interval) is based on the assumed risk in the comparison group and the **relative effect** of the intervention (and its 95% CI).

CI: Confidence interval; **OR:** Odds ratio

GRADE Working Group grades of evidence
High quality: We are very confident that the true effect lies close to that of the estimate of the effect
Moderate quality: We are moderately confident in the effect estimate: The true effect is likely to be close to the estimate of the effect, but there is a possibility that it is substantially different
Low quality: Our confidence in the effect estimate is limited: The true effect may be substantially different from the estimate of the effect
Very low quality: We have very little confidence in the effect estimate: The true effect is likely to be substantially different from the estimate of effect

Table S4: Exploratory one-step individual participant data meta-analysis, proportion of participants experiencing at least one acute respiratory tract infection by baseline vitamin D status categorised by 25 nmol/L strata

Baseline 25(OH)D (nmol/L)	Number of Trials	Proportion with ≥1 ARTI, control group (%)	Proportion with ≥1 ARTI, intervention group (%)	Adjusted odds ratio (95% CI)[1]	P value	P value for interaction
<25	14	137/249 (55.0)	117/289 (40.5)	0.58 (0.40, 0.82)	0.002	0.77
25 to 49.9	19	385/671 (57.4)	459/821 (55.9)	0.98 (0.79, 1.23)	0.89	
50 to 74.9	19	405/631 (64.2)	472/778 (60.7)	0.91 (0.71, 1.16)	0.44	
≥75	16	237/337 (70.3)	248/396 (62.6)	0.68 (0.46, 0.99)	0.05	

1, adjusted for age, sex and study duration. Abbreviations: 25(OH)D, 25-hydroxyvitamin D; ARTI, acute respiratory tract infection; CI, confidence interval.

Table S5: One-step IPDMA, ARTI event rate: sub-group analyses by baseline vitamin D status and dosing regimen.

	N Trials	N Individuals	Rate of ARTI per participant-year, control group	Rate of ARTI per participant-year, intervention group	Adjusted incidence rate ratio (95% CI)[1]	P value	P value for Interaction
**Baseline 25(OH)D (nmol/L)**							
<25	14	509	2.15	1.67	0.78 (0.66, 0.93)	0.004	0.02
≥25	19	3458	2.12	1.91	0.95 (0.90, 1.00)	0.04	
**Dosing Regimen Type**							
Bolus dose ≥30,000 IU given	10	5595	0.73	0.76	0.99 (0.94 to 1.05)	0.83	0.11
Bolus dose not given	15	5133	2.23	2.09	0.93 (0.88 to 0.98)	0.008	

1, adjusted for age, sex and study duration. Abbreviations: 25(OH)D, 25-hydroxyvitamin D; ARTI, acute respiratory tract infection; CI, confidence interval.

Table S6: One-step IPDMA, time to first ARTI: sub-group analyses by baseline vitamin D status and dosing regimen.

	N Trials	N Individuals	Median time to first ARTI, control group, days (IQR)	Median time to first ARTI, intervention group, days (IQR)	Adjusted hazard ratio (95% CI)[1]	P value	P value for Interaction
**Baseline 25(OH)D (nmol/L)**							
<25	10	229	159 (56 to --)	172 (74 to --)	0.92 (0.66 to 1.28)	0.62	0.61
≥25	12	2231	104 (41 to 280)	110 (40 to 328)	0.97 (0.88 to 1.06)	0.48	
**Dosing Regimen Type**							
Bolus dose ≥30,000 IU given	8	4795	-- (121 to --)	-- (117 to --)	0.98 (0.89 to 1.08)	0.74	0.30
Bolus dose not given	10	4313	138 (57 to 331)	153 (61 to 351)	0.91 (0.84 to 0.99)	0.04	

1, adjusted for age, sex and study duration. Abbreviations: --, these values cannot be defined; 25(OH)D, 25-hydroxyvitamin D; ARTI, acute respiratory tract infection; CI, confidence interval.

Table S7: Responder analysis, one-step IPDMA

	N trials	Proportion with >=1 ARTI, (%)	Adjusted odds ratio (95% CI)[1]	P value
Intervention, end-study 25(OH)D < 75 nmol/L	18	542/1120 (48.4)	1	--
Intervention, end-study 25(OH)D ≥ 75 nmol/L	18	784/1291 (60.7)	0.96 (0.78, 1.18)	0.68
	**N trials**	**Median time to first ARTI, days (IQR)**	**Adjusted hazard ratio (95% CI)**	**P value**
Intervention, end-study 25(OH)D < 75 nmol/L	11	190 (63, --)[2]	1	--
Intervention, end-study 25(OH)D ≥ 75 nmol/L	12	102 (39, 312)	1.02 (0.88, 1.19)	0.76
	**N trials**	**Rate of ARTI per participant-year**	**Adjusted incidence rate ratio (95% CI)**	**P value**
Intervention, end-study 25(OH)D < 75 nmol/L	18	1.51	1	--
Intervention, end-study 25(OH)D ≥ 75 nmol/L	18	2.04	1.01 (0.94, 1.10)	0.72

1, adjusted for age, sex and study duration. 2, 75th centile for time to first ARTI in this group cannot be defined. Abbreviations: 25(OH)D, 25-hydroxyvitamin D; ARTI, acute respiratory tract infection; CI, confidence interval; IQR, inter-quartile range.

Figure S1: Cates plots illustrating reduction in risk of acute respiratory tract infection with vitamin D supplementation, irrespective of dosing frequency, in A) all participants, irrespective of baseline vitamin D status, B) participants with baseline serum 25(OH)D concentration <25 nmol/L, and C) participants receiving daily or weekly vitamin D supplementation regimens without any additional bolus doses.

A.

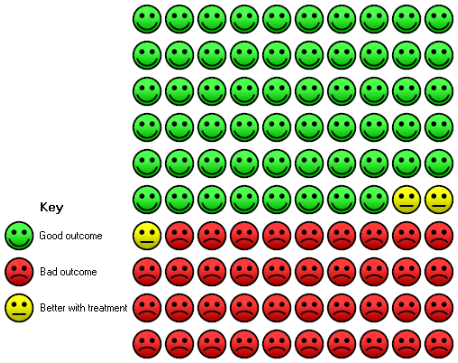

B.

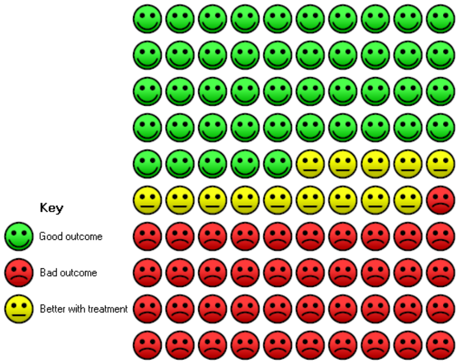

C.

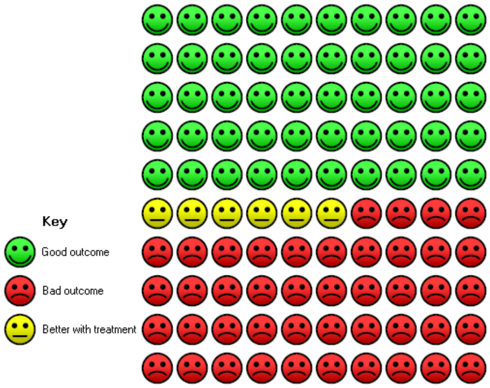

Figure S2: Mean baseline serum 25(OH)D concentration at enrolment by dosing regimen. 'Bolus', studies in which a bolus dose of ≥30,000 IU vitamin D was given in the intervention arm; 'No bolus', studies in which vitamin D was administered daily or weekly without administration of such a bolus dose of vitamin D.

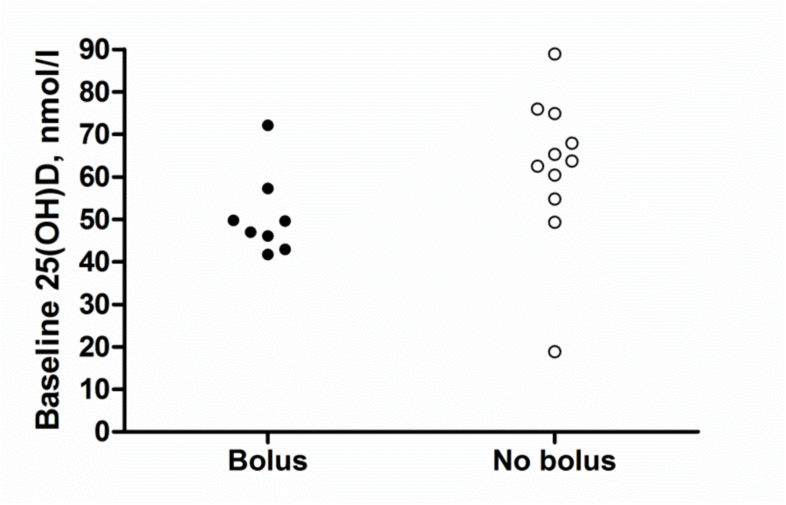

Figure S3: Mean age at enrolment by dosing regimen. 'Bolus', studies in which a bolus dose of ≥30,000 IU vitamin D was given in the intervention arm; 'No bolus', studies in which vitamin D was administered daily or weekly without administration of such a bolus dose of vitamin D.

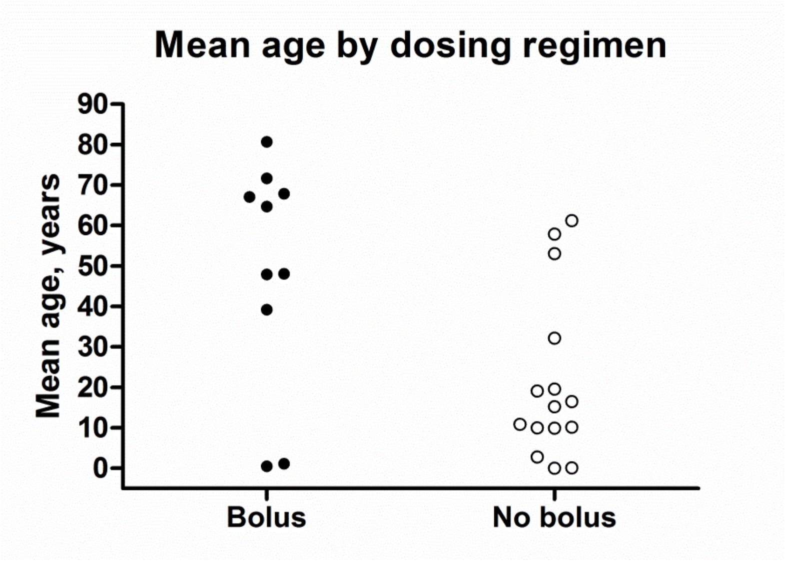

Figure S4: Cates plot illustrating reduction in risk of acute respiratory tract infection with daily/weekly vitamin D supplementation without additional bolus doses in A) participants with baseline serum 25(OH)D concentration <25 nmol/L, and B) participants with baseline serum 25(OH)D concentration ≥25 nmol/L.

A.

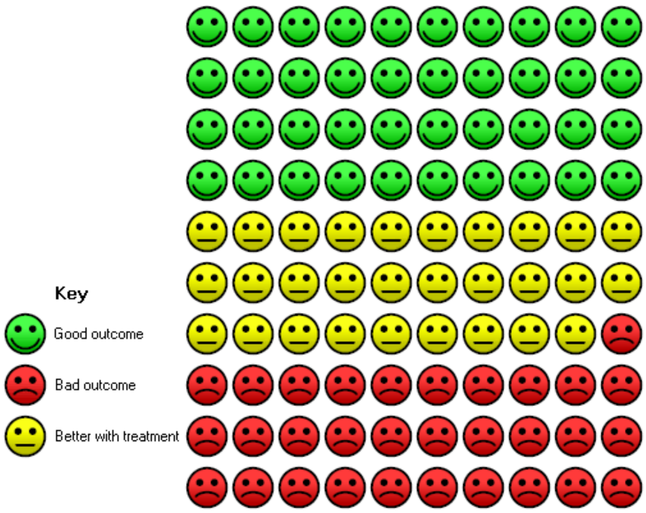

B.

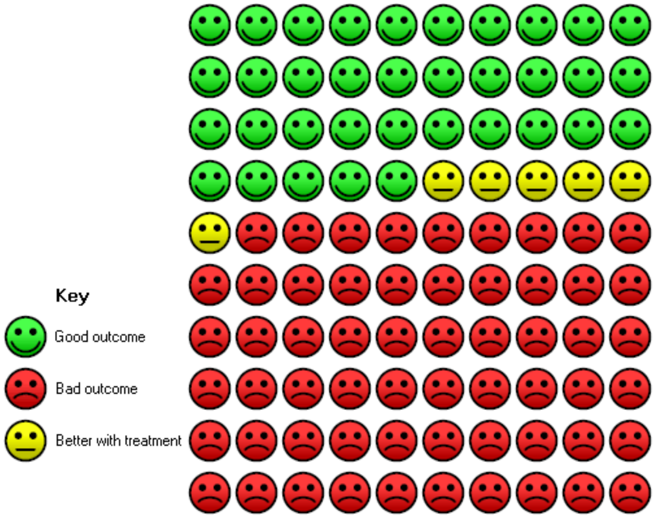

Figure S5: Funnel plot for individual patient data meta-analysis of proportion of participants experiencing at least one acute respiratory tract infection

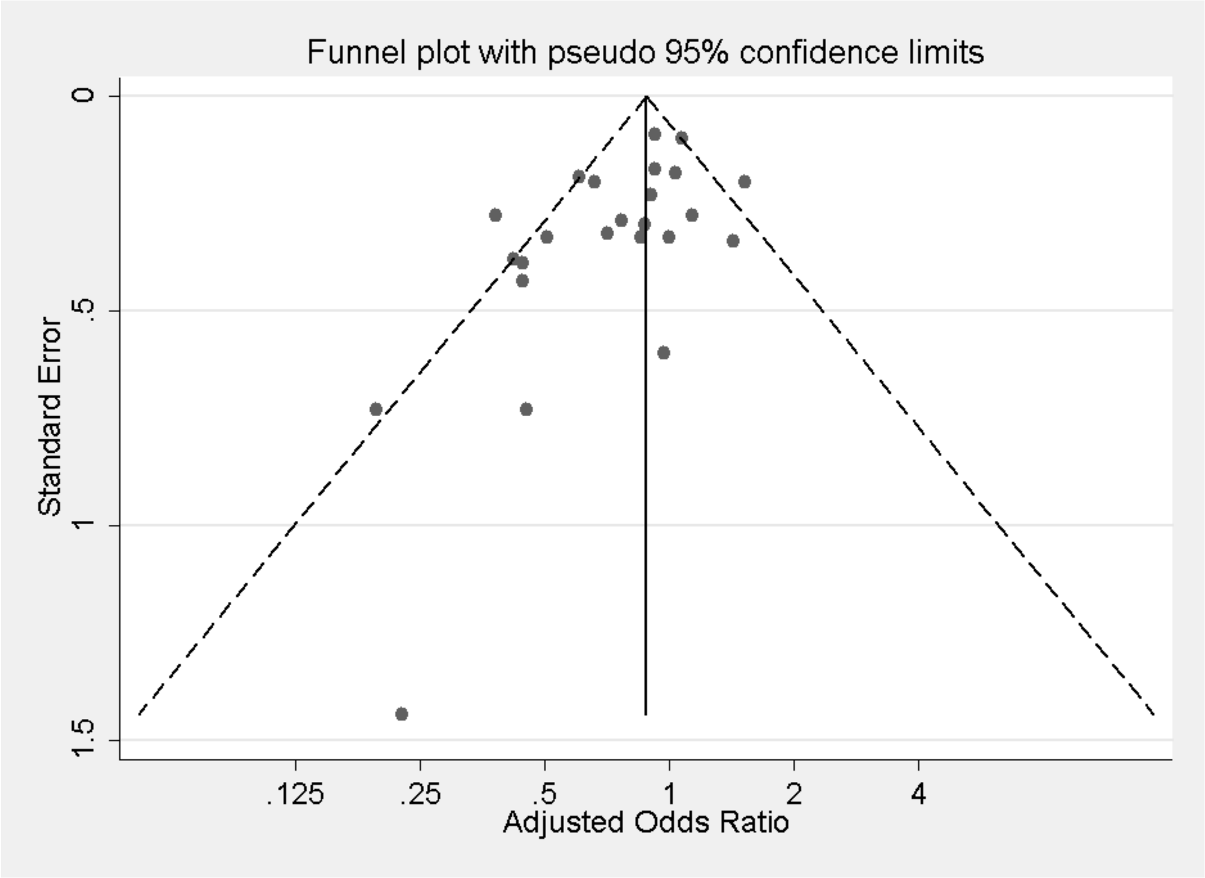

References

1. Li-Ng M, Aloia JF, Pollack S, Cunha BA, Mikhail M, Yeh J, et al. A randomized controlled trial of vitamin D3 supplementation for the prevention of symptomatic upper respiratory tract infections. Epidemiol Infect. 2009; **137**(10): 1396-404.
2. Urashima M, Segawa T, Okazaki M, Kurihara M, Wada Y, Ida H. Randomized trial of vitamin D supplementation to prevent seasonal influenza A in schoolchildren. Am J Clin Nutr. 2010; **91**(5): 1255-60.
3. Manaseki-Holland S, Qader G, Isaq Masher M, Bruce J, Zulf Mughal M, Chandramohan D, et al. Effects of vitamin D supplementation to children diagnosed with pneumonia in Kabul: a randomised controlled trial. Trop Med Int Health. 2010; **15**(10): 1148-55.
4. Laaksi I, Ruohola JP, Mattila V, Auvinen A, Ylikomi T, Pihlajamaki H. Vitamin D supplementation for the prevention of acute respiratory tract infection: a randomized, double-blinded trial among young Finnish men. J Infect Dis. 2010; **202**(5): 809-14.
5. Majak P, Olszowiec-Chlebna M, Smejda K, Stelmach I. Vitamin D supplementation in children may prevent asthma exacerbation triggered by acute respiratory infection. J Allergy Clin Immunol. 2011; **127**(5): 1294-6.
6. Kumar GT, Sachdev HS, Chellani H, Rehman AM, Singh V, Arora H, et al. Effect of weekly vitamin D supplements on mortality, morbidity, and growth of low birthweight term infants in India up to age 6 months: randomised controlled trial. BMJ. 2011; **342**: d2975.
7. Lehouck A, Mathieu C, Carremans C, Baeke F, Verhaegen J, Van Eldere J, et al. High doses of vitamin D to reduce exacerbations in chronic obstructive pulmonary disease: a randomized trial. Ann Intern Med. 2012; **156**(2): 105-14.
8. Manaseki-Holland S, Maroof Z, Bruce J, Mughal MZ, Masher MI, Bhutta ZA, et al. Effect on the incidence of pneumonia of vitamin D supplementation by quarterly bolus dose to infants in Kabul: a randomised controlled superiority trial. Lancet. 2012; **379**(9824): 1419-27.
9. Camargo CA, Jr., Ganmaa D, Frazier AL, Kirchberg FF, Stuart JJ, Kleinman K, et al. Randomized trial of vitamin D supplementation and risk of acute respiratory infection in Mongolia. Pediatrics. 2012; **130**(3): e561-7.
10. Murdoch DR, Slow S, Chambers ST, Jennings LC, Stewart AW, Priest PC, et al. Effect of vitamin D3 supplementation on upper respiratory tract infections in healthy adults: the VIDARIS randomized controlled trial. JAMA. 2012; **308**(13): 1333-9.
11. Bergman P, Norlin AC, Hansen S, Rekha RS, Agerberth B, Bjorkhem-Bergman L, et al. Vitamin D3 supplementation in patients with frequent respiratory tract infections: a randomised and double-blind intervention study. BMJ Open. 2012; **2**(6): e001663.
12. Marchisio P, Consonni D, Baggi E, Zampiero A, Bianchini S, Terranova L, et al. Vitamin D supplementation reduces the risk of acute otitis media in otitis-prone children. Pediatr Infect Dis J. 2013; **32**(10): 1055-60.
13. Rees JR, Hendricks K, Barry EL, Peacock JL, Mott LA, Sandler RS, et al. Vitamin D3 Supplementation and Upper Respiratory Tract Infections in a Randomized, Controlled Trial. Clin Infect Dis. 2013.
14. Tran B, Armstrong BK, Ebeling PR, English DR, Kimlin MG, van der Pols JC, et al. Effect of vitamin D supplementation on antibiotic use: a randomized controlled trial. Am J Clin Nutr. 2014; **99**(1): 156-61.
15. Goodall EC, Granados AC, Luinstra K, Pullenayegum E, Coleman BL, Loeb M, et al. Vitamin D3 and gargling for the prevention of upper respiratory tract infections: a randomized controlled trial. BMC infectious diseases. 2014; **14**: 273.
16. Urashima M, Mezawa H, Noya M, Camargo CA, Jr. Effects of vitamin D supplements on influenza A illness during the 2009 H1N1 pandemic: a randomized controlled trial. Food & function. 2014; **5**(9): 2365-70.

17. Grant CC, Kaur S, Waymouth E, Mitchell EA, Scragg R, Ekeroma A, et al. Reduced primary care respiratory infection visits following pregnancy and infancy vitamin D supplementation: a randomised controlled trial. Acta Paediatr. 2014.
18. Martineau AR, James WY, Hooper RL, Barnes NC, Jolliffe DA, Greiller CL, et al. Vitamin D3 supplementation in patients with chronic obstructive pulmonary disease (ViDiCO): a multicentre, double-blind, randomised controlled trial. The lancet Respiratory medicine. 2015; **3**(2): 120-30.
19. Martineau AR, MacLaughlin BD, Hooper RL, Barnes NC, Jolliffe DA, Greiller CL, et al. Double-blind randomised placebo-controlled trial of bolus-dose vitamin D3 supplementation in adults with asthma (ViDiAs). Thorax. 2015; **70**(5): 451-7.
20. Martineau AR, Hanifa Y, Witt KD, Barnes NC, Hooper RL, Patel M, et al. Double-blind randomised controlled trial of vitamin D3 supplementation for the prevention of acute respiratory infection in older adults and their carers (ViDiFlu). Thorax. 2015.
21. Simpson SJ, van der Mei I, Stewart N, Blizzard L, Tettey P, Taylor B. Weekly cholecalciferol supplementation results in significant reductions in infection risk among the vitamin D deficient: results from the CIPRIS pilot RCT. BMC Nutrition. 2015; **1**(7).
22. Dubnov-Raz G, Rinat B, Hemila H, Choleva L, Cohen AH, Constantini NW. Vitamin D supplementation and upper respiratory tract infections in adolescent swimmers: a randomized controlled trial. Pediatric exercise science. 2015; **27**(1): 113-9.
23. Denlinger LC, King TS, Cardet JC, Craig T, Holguin F, Jackson DJ, et al. Vitamin D Supplementation and the Risk of Colds in Patients with Asthma. Am J Respir Crit Care Med. 2016; **193**(6): 634-41.
24. Tachimoto H, Mezawa H, Segawa T, Akiyama N, Ida H, Urashima M. Improved Control of Childhood Asthma with Low-Dose, Short-Term Vitamin D Supplementation: A Randomized, Double-Blind, Placebo-Controlled Trial. Allergy. 2016.
25. Ginde AA, Blatchford P, Breese K, Zarrabi L, Linnebur SA, Wallace JI, et al. High Dose Monthly Vitamin D for Prevention of Acute Respiratory Infection in Older Long-Term Care Residents: A Randomized Clinical Trial. J Am Geriatr Soc 2016 Nov 16 [Epub ahead of print];]
